# De Witt County Medical Society

**Published:** 1862-05

**Authors:** B. K. Shurtleff

**Affiliations:** Secretary


### DE WITT COUNTY MEDICAL SOCIETY.

The society met in annual session at the office of Dr. W. W. Adams.

The President, Dr. Z. H. Madden in the chair. The minutes of the previous meeting were read and approved.

On motion of Dr. Adams, Dr. Brown of Macon county, was invited to participate in the proceedings of this meeting. On motion the election of officers was postponed until the afternoon session. Essays were called for. Dr. T. K. Edmiston was excused from reading on account of sore eyes; but made a verbal report of a very interesting case of abscess of the liver. He also gave notice that he would read a paper on the formation and treatment of abscesses at the next meeting of the society. Dr. Shurtleff read an essay on diptheria in which he contended that it was a constitutional disease. This gave rise to a very interesting discussion; some of the members contended that it was only local. On motion, the society adjourned to meet at 2 o'clock, P. M.

### AFTERNOON SESSION.

Election of officers being in order the following were duly elected:

John Wright, M. D.—*President.*
Dr. J. R. Richards— *Vice-President.*
Dr. R. T. Richards—*Treasurer.*
Dr. B. K. Shurtleff—*Secretary.*

B. S. Lewis, M. D., T. W. Davis, M. D., and W. W. Adams, M. D.—*Censors.*

Dr. Z. H. Madden, the retiring President, then delivered an excellent valedictory address which was listened to with marked attention. On motion, the address was ordered to be published in the *Central Transcript.* Dr. Brown verbally reported a very peculiar case in a child: The disease first making its appearance by swelling of the scrotum followed with fever, general depression and dark spots from the size of a dime to that of a bottom of a large tea-cup, having the appearance of extravasated blood. The doctor also spoke of four or five similar cases treated by physicians of Decatur and almost invariably terminating fatally.

Scalds and burns, the subject for general debate, was then discussed by most of the members present. Drs. W. W. Adams and D. W. Edmiston were appointed essayists for the next meeting. Mercurials in the treatment of disease was chosen as the subject for discussion at the next meeting of the society. Dr. R. T. Richards offered the following resolution which was unanimously adopted:

*Resolved*, that the thanks of this society are due and hereby tendered to Dr. Madden and lady for the excellent dinner prepared for the members of the society.

On motion, it was ordered that the proceedings of this meeting be published in the *Central Transcript* and Chicago MEDICAL JOURNAL.

On motion, the society adjourned to meet in quarterly session at Waynesville, on the first Tuesday in July next.

DR. B. K. SHURTLEFF, *Secretary.*

---

☞ Subscribers will please direct business or money letters to Dr. Ingals, Box 14, Chicago, Ills.

